# *Ignatzschineria indica* bacteremia in a patient with a maggot-infested heel ulcer: a case report and literature review

**DOI:** 10.1099/acmi.0.000078

**Published:** 2019-11-15

**Authors:** Vincent Deslandes, Colleen Haney, Kathryn Bernard, Marc Desjardins

**Affiliations:** ^1^​ University of Ottawa, Department of Medical Microbiology, The Ottawa Hospital, Ottawa, Canada; ^2^​ University of Ottawa, Department of Surgery, Pembroke Regional Hospital, Pembroke, Ottawa, Canada; ^3^​ National Microbiology Laboratory, Health Canada, Winnipeg, Canada; ^4^​ Division of Microbiology, Department of Pathology and Laboratory Medicine, The Ottawa Hospital, Ottawa, Canada

**Keywords:** *Ignatzschineria indica*, bacteremia, maggots

## Abstract

An elderly patient was admitted with sepsis to a community hospital. The individual was found to have a foot wound infested with maggots, and clinical evidence of cellulitis. A blood culture was positive on day 2 with *Ignatzschineria indica *. The patient was treated successfully with a combination of antibiotics and manual removal of the maggots. Poor living conditions were central to his presentation.

## Introduction

*Ignatzschineria* species are emerging human pathogens within the Gammaproteobacteria that have been recovered from clinical samples from patients with wound myiasis. To date, four species of these Gram-negative, asaccharolytic bacilli have been described: the type species *I. larvae *, *I. indica *, *I. ureiclastica* and the recently described *I. cameli* [1]. Recent inclusion of a reference MS spectra for a representative strain of *I. indica* may lead to more frequent identification of this organism in the future [2] We describe a case of bacteremia due to *I. indica* in a patient with a chronic wound colonized with maggots and provide a literature review of previously reported cases of *I. indica* infections. We provide antimicrobial susceptibility data for two historical Canadian isolates and review prior reports, which seem to indicate that *I. indica* may possess or can acquire resistance to antimicrobials, including broad-spectrum beta-lactams.

## Case

An elderly patient was brought into a community hospital by EMS following a fall from a chair at home. On site, squalid living conditions had been noted, with garbage and rats seen in the house. The patient had vomited once and was covered in faeces. On arrival to the hospital, the individual had a septic picture with hypotension (BP 89/70) and tachycardia (HR 152). On history, the patient acknowledged a poor appetite the previous week, but denied fever, chills or shortness of breath. On examination, a left foot wound covered with innumerable maggots was found, with evidence of cellulitis extending to the mid-lower left leg and excoriation of the toes and forefoot. While the patient indicated that this was secondary to a trauma involving a bedframe a few days prior, the appearance of the wound was in keeping with a chronic process. The individual was a known diabetic with poor sugar control as evidenced by a random glucose level of 18.8 mmol l^−1^ and had had an episode of myocardial infarction 12 years prior. In keeping with the septic picture, the white blood cell count (18.75×10^9^ l^−1^) and blood lactate level (11.2 mmol l^−1^) were elevated. The patient also had an acute kidney injury (creatinine 206 µmol l^−1^), the etiology of which was felt to be prerenal. The individual denied any abdominal discomfort, shortness of breath, urinary symptoms or headaches. The patient was fluid resuscitated with 2l of normal saline. One set of blood cultures (aerobic and anaerobic bottles) was drawn, and empirical treatment with piperacillin-tazobactam was initiated. The foot wound was irrigated with water and then hydrogen peroxide in the emergency department to remove the bulk of the maggots.

**Fig. 1.**
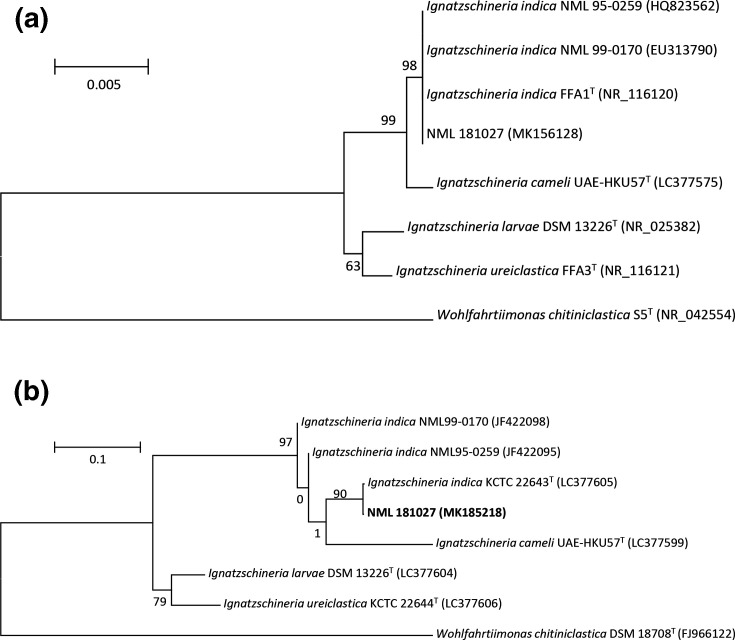
Phylogenetic consensus trees based on nearly full-length 16S rRNA gene sequences (a) or partial *gyr*B sequences (b) for the patient isolate NML 181027 and two historical NML isolates. Alignment done using Clustal W (default settings) and the neighbour-joining (1000 replicates) algorithm found in mega 6.06. A type strain of *Wohlfahrtiimonas chitiniclastica* is used as an unrelated outgroup. Scale infers 0.005 (16S) and 0.1 (*gyr*B) substitutions per nucleotide position.

The aerobic blood culture bottle was flagged positive for growth at the 36 h mark. Gram-variable bacilli were initially seen on direct microscopy and reported as Gram-positive bacilli. The blood was inoculated to solid media plates for bacterial isolation, and small colonies were noted on the surface of blood agar, chocolate and MacConkey plates 48 h post-inoculation. There was no growth on the anaerobic CDC plate. Direct MALDI-ToF-MS analysis (Bruker, Daltonics) on a representative isolated colony returned a positive identification as *I. indica* with a score of 2.47 (Biotyper 7854). Based on this identification, the Gram stain was reviewed and a corrected report for Gram-negative bacilli was issued. The identification was characterized in Ottawa by partial sequencing of the 16s rRNA gene [3] and an initial antimicrobial panel consisting of ceftazidime, ciprofloxacin, meropenem, piperacillin/tazobactam and trimethoprim-sulfamethoxazole was performed by Etest (bioMérieux, Montréal) and read after 24 h incubation at 35C in O_2_ incubator. The isolate was noted to be susceptible to all antibiotics tested. It was subsequently submitted to the Public Health Agency of Canada’s National Microbiology Laboratory (NML) for 16s rRNA [4] and *gyr*B [5] genes sequencing to further confirm the identification. Based on this analysis, it was found to cluster closely with two other strains previously submitted to NML Special Bacteriology laboratory (NML95-0259, NML99-0170) and the type strain *I. indica* FFA1^T^ (Fig. 1a, b), with 100 % similarity among sequences over 1450 and 565 bp, respectively. The two historical NML strains had previously been isolated from blood cultures, and reported as presumptively belonging to Gilardi Rod Group 1 based on biochemical and cellular fatty acid profiles [6]. GenBank accession numbers assigned to NML 181027 are MK156128 and MK185218 for 16S rRNA and *gyr*B gene sequences, respectively. There was also 99.5 % sequence identity with the recently described *I. cameli* type strain UAE-HKU57 [1], but *I. indica* and *I. cameli* isolates could be separated with confidence based on *gyrB* sequencing: strain NML181020 showed >95.1 % sequence similarity with other *I. indica* strains but <87 % similarity with other *Ignatzschineria* species including *I. cameli* (Fig. 1b). This is in keeping with findings by Tsang *et al*. [1]. Results for AST testing at the NML (Table 1) reiterated susceptibility to 15 antimicrobials, using CLSI methods/guidelines [7] for broth microdilution using Sensititre plates and broths (Thermofisher).

**Table 1.** MIC (Sensititre GN2F, ThermoFisher) of various antimicrobials versus *I. indica* stains NML181027 (this study), NML 990170 and NML 95259 with interpretation (CLSI M100 28th Ed, Table 2B–5) and previously reported susceptibility profiles of the type strain FFA1^T^ and other clinical isolates of *I. indica
*

Strains	NML18-1027		NML99-0170		NML95-259		FFA1^T^	Barker *et al*.	Mejjas *et al*.
Methodology	Sensititre		Sensititre		Sensititre		DD	n/a	Vitek 2
Antibiotics	MIC	INT	MIC	INT	MIC	INT	INT	INT	INT
AMK	<8	S	16	S	16	S	S		S
AMP	<4	–	>32	–	<4	–	**R**		
SAM	<4/2	–	>32/16	–	8/4	–			
ATM	<8	S	**>32**	**R**	<8	S	**R**	S	S
CFZ	<4	–	>32	–	<4	–			
FEP	<4	S	**>32**	**R**	**32**	**R**		S	
CTT	<8	–	>32	–	<8	–			
FOX	<4	–	>32	–	16	–	**I**		
CPD	<2	–	>16	–	<2	–			
CRO	<1	S	**>64**	**R**	2	S	S	S	
CAZ	2	S	**>32**	**R**	**>32**	**R**	**I**		S
CRX	<4	–	>32	–	8	–			
CIP	<0.5	S	<0.5	S	<0.5	S	S	**I**	S
GAT	<1	S	<1	S	<1	S			
GEN	<2	S	<2	S	<2	S	S	S	
IMP	<2	S	**>16**	**R**	<2	S			
MEM	<1	S	**>8**	**R**	<1	S		S	S
NIT	<16	–	<16	–	<16	–	S		
PIP	<16	S	**>128**	**R**	**32**	**I**			
TZP	<16/4	S	**>128/4**	**R**	**32/4**	**I**	S		
TIM	<16/2	S	**>64/2**	**R**	<16/2	S	**R**		
SXT	<0.5/9.5	S	<0.5/9.5	S	<0.5/9.5	S	**I**	S	S
TOB	<4	S	<4	S	<4	S	S	S	S

S: susceptible, R: resistant, I: intermediate, -: no interpretation, AMK: amikacin, AMP: ampicillin, SAM: ampicillin-sulbactam, ATM: aztreonam, CFZ: cefazolin, FEP: cefepime, CTT: cefotetan, FOX: cefoxitin, CPD: cefpodoxime, CRO: ceftriaxone, CAZ: ceftazidime, CRX: cefuroxime, CIP: ciprofloxacin, GAT: gatifloxacin, GEN: gentamicin, IPM: imipenem, MEM: meropenem, NIT: nitrofurantoin, PIP: piperacillin, TZP: piperacillin-tazobactam, TIM: ticarcilin-clavulanic acid, SXT: trimethoprim-sulfamethoxazole, TOB: tobramycin, DD: disk diffusion, n/a: data not available

**Fig. 2.**
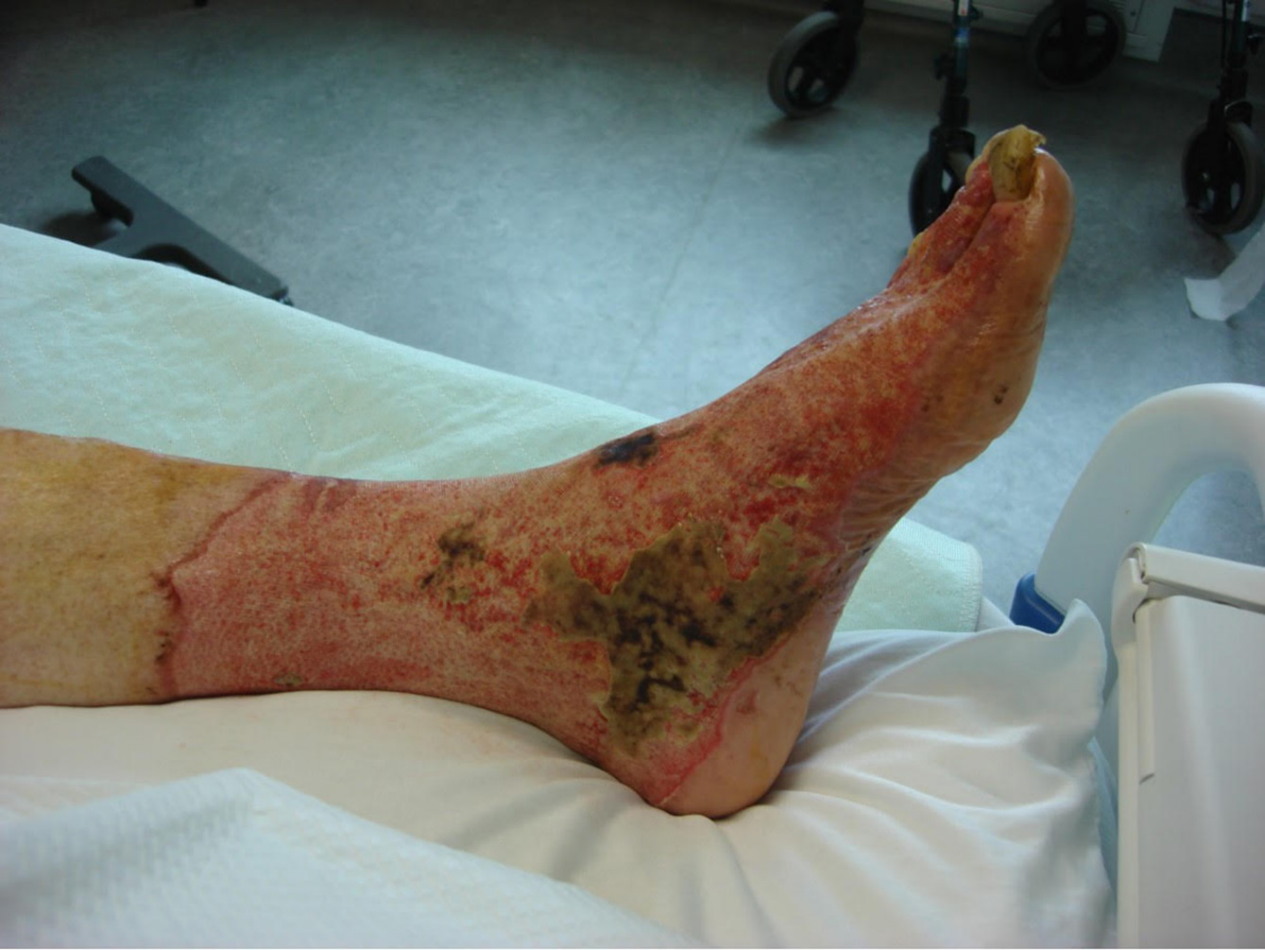
Patient’s left foot, post-admission day 4, after multiple sessions of debridement and maggot removal. Cellulitis has resolved at this point, with some remaining areas of necrotic tissue but no visible maggot. Patient consent was obtained for photography and publication.

The patient was treated with piperacillin-tazobactam for 7 days, and then transitioned to oral amoxicillin-clavulanic acid for a further 7 days. The foot wound required daily manual maggot removal and providone baths. By admission day 3, the cellulitis had started to settle, and by admission day 4 there was no further evidence of ongoing maggot infestation (Fig. 2.). The patient’s wound improved throughout their hospital stay, and no recurrence of myiasis was noted during hydroalginate dressing changes every other day. The individual was discharged from the hospital on admission day 14 with home wound care services for dressing changes. They were re-admitted to the hospital a week later for reasons unrelated to the initial infectious presentation and remained hospitalized for a month. No further antibiotherapy was required during that time. By the end of this second admission, the foot wound had fully healed.

## Discussion

*I. indica* is a non-motile, non-haemolytic, non-sporulating, catalase positive, oxidase positive, aerobic Gram-negative bacilli, and can be recovered in the clinical microbiology laboratory from specimens inoculated on routine isolation media like blood agar or MacConkey agar [8]. It is phylogenetically unassigned to a Family within the Gammaproteobacteria [9] and was first recovered from the gut content of adult flesh flies, as were other species of the *Ignatzschineria* genus: *I. larvae* (type species of the genus)*, I. ureiclastica* and the recently described *I. cameli* [1]. Along with *W. chitiniclastica *, another maggot-associated bacteria that has caused episodes of bacteremia, these organisms have been referred to as emerging pathogens [10–12]. Recovery of *I. indica* from clinical specimens has only been documented in the past decade, and with increased frequency in the past few years. However, the paucity of case reports related to *Ignatzschineria* species may also be a reflexion of the lack of reliable means to identify them until recently. The strain here was correctly identified by MALDI-ToF MS.

To date, there have been four case reports and one case collection describing *I. indica* from seven patients [10, 13–16], six of which originated from the USA and one from Argentina. All had chronic non-healing wounds. Five patients had obvious wound myiasis with concomitant positive blood cultures with *I. indica *, and all these patients had favourable outcomes with antibiotherapy. This is in contrast with results presented in a recent review of *W. chitiniclastica* cases where two out of four patients with bacteremia did not survive [11]. In three cases of *I. indica* bacteremia the blood cultures were polymicrobial and included pathogens such as *Providencia stuartii *, *Streptococcus pyogenes, Streptococcus anginosus* and *W. chitiniclastica* [13, 15, 16], reflecting the complex, likely polymicrobial nature of wounds colonized by maggots. *I. indica* was misidentified on two occasions; it was mistakenly identified as *Alcaligenes faecalis* by RapidID NF Plus, and as *Acinetobacter* sp. by Vitek 2 prior to formal identification by 16s rRNA gene sequencing [13]. Of note, one case documented lack of identification by MALDI-ToF-MS (Bruker, Daltonics), although the version of the database used was not specified [10]. Reference spectra for one strain of *I. indica* has since been added to the Bruker database in 2016 [2] and as such it is unclear whether identification of this genus down to the species level is reliable. Our strain failed to generate an identification with Vitek MS (version 3.0).

*I. indica* had initially only been associated with *Wohlfahrtia magnifica*, which can also carry *W. chitiniclastica *, and *Sarcophaga* sp., both members of the *Sarcophagidae* family of flesh flies [8], which are seldom found in North America and are known agents of furuncular myiasis. It is therefore striking that all reported clinical cases of wound myiasis with *I. indica* infections originated from the Americas even though the reference *I. indica* strain was isolated in India, hinting at a global distribution of the bacteria. In two reported clinical cases of *I. indica* bacteremia the vector was recovered and identified as *Lucilia (Phaenicia) sericata* [13, 16], the common greenbottle fly, which is used in maggot-debridement therapy [17]. It is widespread in North America and recognized as the agent of sheep strike, a type of myiasis in sheep [18]. Wound myiasis has also previously been associated with *Lucilia* sp. in Canada [19]. To date, there has been no reported cases of *I. indica* septicemia linked to maggot-debridement therapy. The concurrent isolation of both *W. chinitoclastica* and *I. indica* from the bloodstream of a patient with wound myiasis suggest that both can share the same vector [16], but the full range of vectors for these bacteria in the context of human infections has yet to be established. This is further complicated by the fact that maggots were not routinely sent for formal identification in most cases of wound myiasis, including ours.

The strain recovered in this report and characterized by the National Microbiology Laboratory (identifier: NML181027) clustered with other known Canadian *I. indica* strains by 16s rRNA (Fig. 1a) and *gyr*B gene sequencing (Fig. 1b), and exhibited universal susceptibility to common antimicrobials by the broth microdilution method (Sensititre GN2F, ThermoFisher) (Table 1). Previous clinical isolates for which susceptibility testing has been documented exhibited susceptibility to trimethoprim-sulfamethaxole and aminoglycosides, and only one isolate showed intermediate susceptibility to ciprofloxacin [13, 15]. Interestingly, the type strain of *I. indica *, which was not a clinical isolate, was noted to be resistant to ampicillin, amoxicillin-clavulanic acid, cefalotin, colistin, mecillinam, metronidazole, penicillin, teicoplanin, ticarcillin and vancomycin, and had intermediate susceptibility to carbenicillin, ceftazidime, cephalexin, cefoxitin, colistin, trimethoprim-sulfamethoxazole, doxycycline and gatifloxacin by the Kirby–Bauer method. It remained susceptible to ciprofloxacin, aminoglycosides, azithromicin, ceftriaxone, cefotaxime, chloramphenicol, clindamycin, erythromycin, nitrofurantoin, piperacillin/tazobactam and tetracycline [8]. It is unclear which interpretation criteria were used, but this susceptibility profile is similar to that of *I. indica* strain NML99-0170 previously isolated from the blood of a Canadian patient, which also exhibited resistance to carbapenems. This profile, combining resistance to a monobactam (aztreonam), non-susceptibility to third-generation cephalosporins and resistance to carbapenem could be in keeping with the presence of a carbapenemase, or ESBL-type of enzyme with a porin mutation, but could also have evolved from chromosomal mutations in the context of environmental exposure. While no ESBL or carbapenemase enzymes have been documented in *I. indica *, our current data indicate that this organism can acquire or develop resistance to antimicrobials. This is in contrast with strains of *W. chitiniclastica,* which only exhibited resistance to fosfomycin [11]. All documented isolates of *I. indica* exhibited susceptibility to trimethroprim-sulfamethaxole, and only one isolate showed intermediate susceptibility to ciprofloxacin. This suggests that aminoglycosides and fluoroquinolones may be the better options for the treatment of clinical infections. In three out of four cases of bacteremia with *I. indica *, definitive treatment involved the use of a fluoroquinolone, with good clinical outcomes noted [10, 13, 15]. For two of those cases, definitive management also involved amputation of the affected limb, likely in the context of comorbid advanced vascular disease. Fortunately, our patient responded well to therapy with beta-lactams. In comparison, all strains of *I. cameli* were noted to be resistant to TMP/SMX, with intermediate susceptibility noted for fluoroquinolones, piperacillin and cefepime [1].

To the best of our knowledge, this is the first case of *I. indica* bacteremia reported in Canada. All reported cases of bacteremia with *I. indica* have been associated with wound myiasis, and potential vectors for this organism are commonly found in North America. Some clinical isolates have shown resistance to broad-spectrum beta-lactams, with no formal resistance mechanism identified at this time. Poor socioeconomic status and living conditions were noted in all reported literature cases, which likely contribute to contacts with vectors carrying *I. indica *.
